# The suppressive role of miR-542-5p in NSCLC: the evidence from clinical data and in vivo validation using a chick chorioallantoic membrane model

**DOI:** 10.1186/s12885-017-3646-1

**Published:** 2017-09-19

**Authors:** Rong-quan He, Xiao-jiao Li, Lu Liang, You Xie, Dian-zhong Luo, Jie Ma, Zhi-gang Peng, Xiao-hua Hu, Gang Chen

**Affiliations:** 1 Department of Medical Oncology, First Affiliated Hospital of Guangxi Medical University, 6 Shuangyong Road, Nanning, 530021 Guangxi Zhuang Autonomous Region People’s Republic of China; 2 Department of PET-CT, First Affiliated Hospital of Guangxi Medical University, Nanning, Guangxi, Zhuang Autonomous Region People’s Republic of China; 3 Department of Pathology, First Affiliated Hospital of Guangxi Medical University, 6 Shuangyong Road, Nanning, 530021 Guangxi Zhuang Autonomous Region People’s Republic of China

**Keywords:** NSCLC, miR-542-5p, CAM, RT-qPCR, GO, KEGG

## Abstract

**Background:**

Non-small cell lung cancer (NSCLC) has led to the highest cancer-related mortality for decades. To enhance the efficiency of early diagnosis and therapy, more efforts are urgently needed to reveal the origins of NSCLC. In this study, we explored the effect of miR-542-5p in NSCLC with clinical samples and in vivo models and further explored the prospective function of miR-542-5p though bioinformatics methods.

**Methods:**

A total of 125 NSCLC tissue samples were collected, and the expression of miR-542-5p was detected by qRT-PCR. The relationship between miR-542-5p level and clinicopathological features was analyzed. The effect of miR-542-5p on survival time was also explored with K-M survival curves and Cox’s regression. The effect of miR-542-5p on the tumorigenesis of NSCLC was verified with a chick chorioallantoic membrane (CAM) model. The potential target genes were predicted by bioinformatics tools, and relevant pathways were analyzed by GO and KEGG. Several hub genes were validated by Proteinatlas.

**Results:**

The expression of miR-542-5p was down-regulated in NSCLC tissues, and consistent results were also found in the subgroups of adenocarcinoma and squamous cell carcinoma. Down-regulation of miR-542-5p was found to be connected with advanced TNM stage, vascular invasion, lymphatic metastasis and EGFR. Survival analyses showed that patients with lower miR-542-5p levels had markedly poorer prognosis. Both tumor growth and angiogenesis were significantly suppressed by miR-542-5p mimic in the CAM model. The potential 457 target genes of miR-542-5p were enriched in several key cancer-related pathways, such as morphine addiction and the cAMP signaling pathway from KEGG. Interestingly, six genes (GABBR1, PDE4B, PDE4C, ADCY6, ADCY1 and GIPR) from the cAMP signaling pathway were confirmed to be overexpressed in NSCLCs tissues.

**Conclusions:**

This evidence suggests that miR-542-5p is a potential tumor-suppressed miRNA in NSCLC, which has the potential to act as a diagnostic and therapeutic target of NSCLC.

## Background

Non-small cell lung cancer (NSCLC) is the most frequent type of lung cancer with high mortality worldwide [1]. Lung adenocarcinoma (LUAD) and squamous cell carcinoma (LUSC) are the major subtypes of NSCLC, composing approximately 40% and 30% of NSCLC, respectively [2]. Like most malignancies, the patients with NSCLC who received diagnoses at an early stage achieved higher five-year survival rates, compared to patients whose diagnoses were made at an advanced stage [3]. However, only a minority of NSCLC patients received early diagnosis because of the lack of significant symptoms in early stages [3]. Currently, therapeutic measures for advanced NSCLC patients are still limited. Although the study of molecular targeted therapies is progressing, including EGFR and ALK-targeted therapies in lung adenocarcinoma, which have had a successful beginning, they are efficient in just 20% of patients [4]. Given these results, high-performance biological markers are critically needed to find and diagnose NSCLC in early stages, to prevent NSCLC from advancing, and to help advanced patients achieve a better prognosis.

MicroRNAs (miRNAs, miRs) are a type of non-coding RNA with a short (less than 22 nucleotides), single stranded nucleotide chain. MiRNAs can regulate the generation of proteins by binding to the untranslated region of messenger RNAs, using complementary base pairing. Through this mechanism, miRNAs can regulate the differentiation, proliferation and apoptosis of cells [5]. Many studies have found that the dysregulation of miRNAs correlates with diseases, including lung cancer [6, 7]. In NSCLC, hundreds of dysregulated miRNAs have been detected from high-throughput experiments [8, 9]. However, the clinicopathological significance and related mechanisms of dysregulated miRNAs in NSCLC remain largely unclear. In preliminary studies, we found that miR-30a [10], miR-193a-3p and miR-133a-3p were down-regulated in NSCLC tissues [11, 12], and all of them have an effect on survival time of patients.

In the current study, we explored the expression of miR-542-5p in NSCLC tissues, assessed the relationship between miR-542-5p and clinicopathological parameters, and verified the function of miR-542-5p on NSCLC in vivo. Furthermore, the potential mechanism of miR-542-5p action on NSCLC was predicted by bioinformatics methods.

## Methods

### Tissue samples

The tissue samples fixed in this study were from 125 lung cancer patients who underwent surgeries at the First Affiliated Hospital of Guangxi Medical University between January 2012 and February 2014. All tissues were obtained before any cancer-related therapy was carried out. Adjacent noncancerous tissues were obtained from at least two centimeters away from the edge of the tumor node. All samples were prepared in the form of formalin-fixed and paraffin-embedded (FFPE). The included lung cancer tissues were divided into 101 LUAD, 23 LUSC and 1 large cell lung cancer. Among 125 included patients, 75 were males, and 50 were females. There were 57 younger (<60 years) patients and 68 older (>60 years) patients. The subgroups were divided based on clinicopathological parameters, such as tumor size, smoking history, and vascular invasion, which are displayed in Table 1. Among 125 patients, 57 LUAD patients were followed-up until the manuscript deadline; 39 patients were alive, and 18 patients were dead. The study was permitted by the Ethical Committee of the First Affiliated Hospital of Guangxi Medical University. Written informed agreements were obtained from the patients and clinicians for the samples usage. Two pathological doctors reviewed all tissues independently.

**Table 1:** Correlations between miR-542-5p expression and the clinicopathological features of NSCLC

Parameters	n	The expression of miR-542-5p (2^-△Cq^)	t	*p*
Tissue				
NSCLC	125	1.952 ± 1.507	−11.703	< 0.001*
Adjacent lung	125	4.568 ± 1.993		
Age (years)				
< 60	57	1.945 ± 1.382	−0.052	0.958
≥ 60	68	1.959 ± 1.615		
Gender				
Male	75	1.983 ± 1.475	0.279	0.783
Female	50	1.906 ± 1.568		
Tumor size (cm)				
< 3	60	1.979 ± 1.520	0.183	0.504
≥ 3	65	1.928 ± 1.506		
Smoking history^a^				
Positive	30	2.622 ± 1.510	−1.004	0.319
Negative	38	2.266 ± 1.407		
TNM stage				
I-II	54	2.822 ± 1.536	6.488	< 0.001*
III-IV	71	1.292 ± 1.101		
Lymph node metastasis				
Positive	69	1.504 ± 1.266	3.905	< 0.001*
Negative	56	2.506 ± 1.604		
Vascular invasion				
Positive	35	1.475 ± 1.305	2.247	0.026*
Negative	90	2.138 ± 1.545		
EGFR protein^b^				
low	17	0.739 ± 0.407	7.753	< 0.001*
high	40	3.049 ± 1.194		

*n* The number of patients

*p < 0.05

^a^The data were available from 68 patients

^b^The data were available from 57 patients

### RNA extraction and expression of miR-542-5p in NSCLC tissues

For FFPE tissues, five sections were acquired from each tissue sample, at a thickness of 10 μm per section. Total RNA was extracted by the RNeasy FFPE Kit (NO. 73504) as stated by the manufacturer’s instructions. The concentration and purity of total RNA were confirmed with a NanoDrop 2000 spectrophotometer (Wilmington, DE, USA). Complimentary DNA was synthesized by total RNA and the TaqMan MicroRNA Reverse Transcription Kit (NO. 4366596) as described previously [13]. Subsequently, an Applied Biosystems PCR7900 system was used to carry out real-time quantitative reverse transcriptase-polymerase chain reactions (qRT-PCR) to detect and analyze the expression of miR-542-5p. The relative expression of miR-542-5p was calculated by 2^-△Cq^. RNA extraction and qRT-PCR were performed in June of 2014.

### Cell culture and expression of miR-542-5p in NSCLC cells

H460, H1299, PC9 and A549 cell lines were cultured in this study, and all the cells were achieved from the Type Culture Collection of the Chinese Academy of Sciences, Shanghai, China. Cells were cultured in Dulbecco’s modified essential medium (DMEM, Invitrogen Corp., Grand Island, NY, USA). Gentamicin, glutamine and fetal bovine serum (10% heat-inactivated, Invitrogen Crop., Grand Island, NY, USA) were supplied in the culture medium. The environment was set at 5% CO_2_, 37 °C and 100% humidity. The expression of miR-542-5p in cells was detected by qRT-PCR. Total RNA was extracted with the TaKaRa MiniBEST Universal RNA Extraction Kit (NO. 9767). The Mir-X™ miRNA First-Strand Synthesis Kit was used to synthesize cDNA: the total volume of the reaction system was 10 μl, and the parameters were set as follows: 37 °C for 15 min, 85 °C for 5 min, and then 4 °C indefinitely. The PCR reaction components included UItra SYBR Mixture (2X, 10 μl), forward primers (10 μm/μl, 1 μl), reverse primers (10 μm/μl, 1 μl), ddH2O (7 μl) and cDNA (1 μl). After 45 cycles (95 °C for 15 s, 60 °C for 16 s, 72 °C for 20 s), reactions were performed in 96-well plates using the Roche LightCycle 480 Real-Time fluorescence quantitative PCR system. The primers used in qRT-PCR were designed with Prime 3.0 software and synthesized by Invitrogen Company. The primer sequences were designed as follows: miR-542-5p (Forward: 5′-GCGGTCGGGGATCATCATGTC; Reverse: 5′-ATCCAGTGCAGGGTCCGAGG). The relative expression of miR-542-5p was calculated using 2^-△Cq^.

### MiR-542-5p lentivirus construction

A lentivirus vector was constructed according to the information of the miR-542-5p sequence and the Ubi-MCS-SV40-EGFP-IRES-puromycin (GV369) polyclone site. The 293 T cell line and Lipofectamine 2000 (Invitrogen, USA) were used to package the lentivirus. The construction and packaging of miR-542-5p lentivirus vector, as well as lentivirus infection, were performed by Shanghai Genechem Co. Ltd., and stored at −80 °C.

### Cell transfection

Among four types of NSCLC cells, the H460 cell line showed the lowest level of miR-542-5p, so we chose H460 to conduct the following experiments. Blank control, empty vector control (H460-Lv-vector) and experimental (H460-Lv-miR-542) groups were designed. H460 cells were seeded and infected with lentivirus in 6-well plates at an MOI of 100 and selected by puromycin. The green fluorescence expressed in cells served as a marker to measure the infection efficiency.

### Chick chorioallantoic membrane model

The chick chorioallantoic membrane (CAM) model was used to explore the effect of miR-542-5p on tumor growth and angiogenesis of NSCLC cells. After being obtained from local hatchery, fertilized eggs were incubated in an incubator at 37 °C and 80% humidity and rotated every 5 h until day 7. On the 8th day of fertilization, air space and embryo were checked under the egg candler, and the head of the embryo and large vessels in the CAM were marked by pencil. To separate the chorioallantoic membrane from the inner shell membrane, a tiny hole (φ = 0.1 cm) in the middle of the air space was made by a mini electric grinder, and an aurilave was used to draw out the gas. A 1.5 cm^2^ window was made in the shell where large vessels in the CAM were marked. A silicone ring (φ = 0.5 cm, height = 0.3 cm) was put at the crossing of large vessels, and cells were then transferred into the silicone ring; the quantity of cells was 10 × 10^6^ in each egg. The eggs were randomly grouped into blank control, empty vector control (H460-Lv-vector) and experimental (H460-Lv-miR-542) groups. Pictures were taken at 0, 24, 48, 72, 96 and 110 h. After every time pictures were taken, the windows were resealed by transparent tape. The silicone ring was removed at 24 h, and transplant implanted tumors were harvested at 110 h. The vascular density was assessed by Image Pro Plus. The harvested tumors were sliced and examined by hematoxylin and eosin (HE) staining. Immunohistochemical (IHC) staining was used to assess the expression of EGFR, VEGF and D2–40. Specific pathogen free fertilized Guangxi Sanhuang chick eggs were purchased from the Experimental Animal Center of Guangxi Medical University (Guangxi, China) and all procedures involving animals and their care complied with the China National Institutes of Healthy Guidelines for the Care and Use of Laboratory Animals. Ethical approval for the study was granted by the Ethical Committee of the First Affiliated Hospital of Guangxi Medical University.

### Biological informatics analysis of potential target genes of miR-542-5p

The target genes of miR-542-5p were predicted by 13 platforms (miRWalk, Microt4, miRanda, mirbridge, miRDB, miRMap, miRNAMap, Pictar2, PITA, RNA22, RNAhybrid, Targetscan and mirTarbase). Genes co-predicted on at least five platforms were collected. To explore the potential function of miR-542-5p in NSCLC, the target genes were analyzed by Gene Ontology (GO) analysis, the Kyoto Encyclopedia of Genes and Genomes (KEGG) pathways in Gorilla (http://cbl-gorilla.cs.technion.ac.il/) and DAVID (https://david.ncifcrf.gov/). String (http://www.string-db.org/) was used to analyze the connections among genes and draw connected figures. The protein expression data from Proteinatlas (http://www.proteinatlas.org/) was checked for the validation of several potential target genes of miR-542-5p in NSCLCs.

### Statistical analysis

The medium value of miR-542-5p was used to divide the NSCLC patients into low expression and high expression groups. Student’s t test was selected to assess the different expression levels of miR-542-5p between NSCLC and adjacent lung tissues, trials and control groups. Spearman method was used to assess the relationship between miR-542-5p level and clinical pathological parameters. Kaplan-Meier (KM) method, log rank test as well as Cox’s regression were performed in the survival analyses. Adjusted hazard ratios (HRs) were calculated. *p* < 0.05 was considered to be statistically significant. The data were calculated by SPSS 22.0, and figures were constructed with Graphpad Prism 5.0.

## Results

### The expression of miR-542-5p in NSCLC tissues

**Fig. 1:**
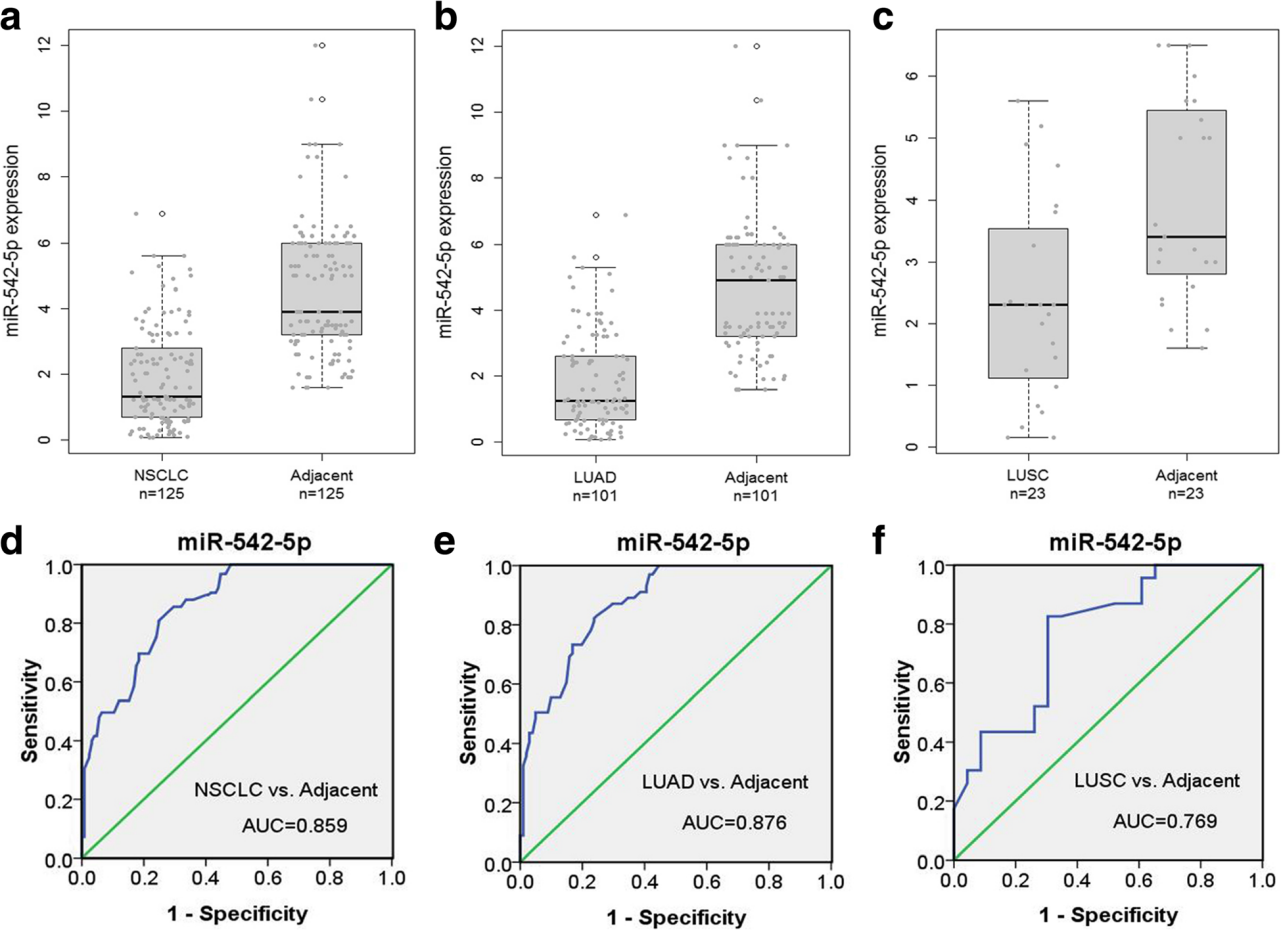
The expression levels of miR-542-5p in NSCLC and adjacent lung tissues. **a**, **b** and **c**: Comparison of miR-542-5p levels in NSCLC (**a**), LUAD (**b**), LUSC (**c**) and adjacent lung tissues; **d**, **e** and **f**: ROC curves of miR-542-5p to predict NSCLC (**d**), LUAD (**e**) and LUSC (**f**)

MiR-542-5p showed evidently lower expression in NSCLC when compared to adjacent normal lung tissues, and consistent results were also found in the subgroups of LUAD and LUSC (Fig. 1a–c). The diagnostic value was assessed by ROC curves. The AUC values were 0.859, 0.876 and 0.769 in NSCLC, LUAD and LUSC, respectively (Fig. 1d–f).

### The relationship between miR-542-5p and clinicopathological features

**Fig. 2:**
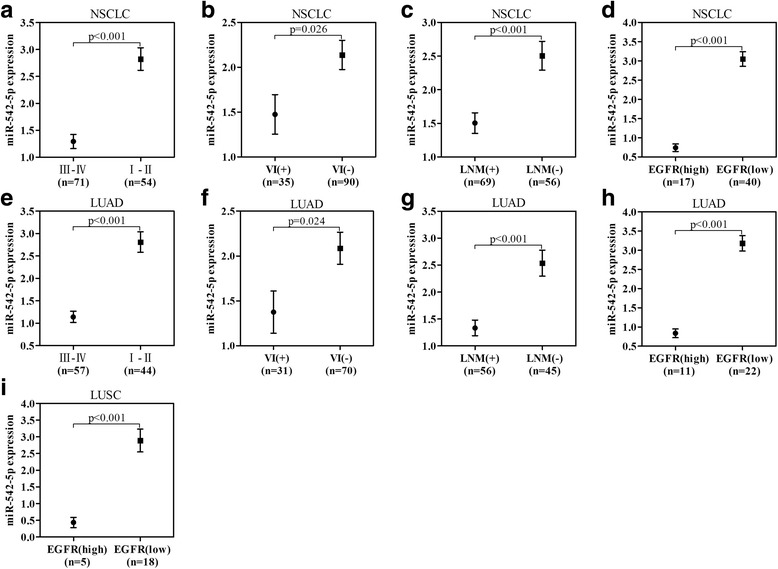
Relative expression of miR-542-5p in different clinicopathological groups of NSCLC patients. **a**, **b**, **c** and **d**: In NSCLC, different expression of miR-542-5p in relative groups are divided by TNM, vascular invasion, lymphatic metastasis and EGFR status; **e**, **f**, **g** and **h**: In LUAD, different expression of miR-542-5p in relative groups are divided by TNM, vascular invasion, lymphatic metastasis and EGFR status; **i**: In LUSC, different expression levels of miR-542-5p in relative groups are divided by EGFR status

In 125 NSCLC patients, down-regulation of miR-542-5p was correlated with advanced TNM stage, vascular invasion and lymphatic metastasis (Fig. 2, Table 1). In TNM I-II stages, the miR-542-5p level was 2.822 ± 1.536, prominently higher than that in III-IV stages (1.292 ± 1.101, *t* = 6.488, *p* < 0.001). Of patients without lymph node metastasis, the miR-542-5p level was clearly overexpressed than those with lymph node metastasis (2.506 ± 1.604 vs 1.504 ± 1.266, *t* = 3.905, *p* < 0.001). Similarly, significantly higher expression of miR-542-5p was also found in the samples without vascular invasion than that with invasion (2.138 ± 1.545 vs 1.475 ± 1.305, *t* = 2.247, p < 0.001). Spearman’s correlation analyses showed that the expression of miR-542-5p was negatively correlated with TNM stage (*r* = −0.505, p < 0.001), lymph node metastasis (*r* = −0.332, p < 0.001), and vascular invasion (*r* = −0.199, *p* = 0.026). In subgroups, down-regulation of miR-542-5p was also found to be correlated with advanced TNM stage, vascular invasion, lymphatic metastasis and EGFR expression in LUAD patients (Fig. 2). In 101 LUAD cases, the expression of miR-542-5p in stage III-IV patients was significantly lower than that of patients at stage I and II (1.142 ± 0.935 vs 2.810 ± 1.516, *t* = 6.417, *p* < 0.001). MiR-542-5p expression in patients with vascular invasion was significantly lower than that in patients without vascular invasion (1.375 ± 1.30 vs 2.087 ± 1.497, *t* = 2.286, *p* = 0.024). And miR-542-5p expression in patients with lymph node metastasis was significantly lower than that in patients without lymph node metastasis (1.333 ± 1.095 vs 2.085 ± 1.085, *t* = −2.477, *p* = 0.027). Additionally, the expression of miR-542-5p was higher in patients who smoke than that of patients without smoking (3.065 ± 1.542 vs 2.535 ± 1.616, *t* = 4.265, *p* < 0.001). Spearman’s correlation analyses showed that the expression of miR-542-5p was negatively correlated with TNM stage (*r* = −0.564, *p* < 0.001), lymph node metastasis (*r* = −0.408, *p* < 0.001), and vascular invasion (*r* = −0.224, p = 0.024). In 23 LUSC cases, there was no significant association between the expression of miR-542-5p and clinical parameters.

In tumor tissues of NSCLC patients, the relative expression of miR-542-5p in patients with high EGFR protein expression was significantly lower than that of patients with low EGFR protein expression (0.739 ± 0.407 vs 3.049 ± 1.194, *t* = 7.753, *p* < 0.001). Spearman’s correlation analyses showed that the expression of miR-542-5p was negatively correlated with EGFR protein expression (*r* = −0.723, p < 0.001). While in the groups of LUAD and LUSC, the expression of miR-542-5p in patients with high EGFR protein expression was significantly lower than that of patients with low EGFR protein expression (0.836 ± 0.373 vs 3.180 ± 0.952, *t* = 10.098, *p* < 0.001, LUAD; 0.436 ± 0.345 vs 2.888 ± 1.448; *t* = 6.543, p < 0.001, LUSC). Spearman’s correlation analyses also showed that the expression of miR-542-5p was negatively correlated with EGFR protein expression in LUAD (*r* = −0.818, *p* < 0.001) and LUSC (*r* = −0.828, p < 0.001, Fig. 2).

### Survival analysis

**Fig. 3:**
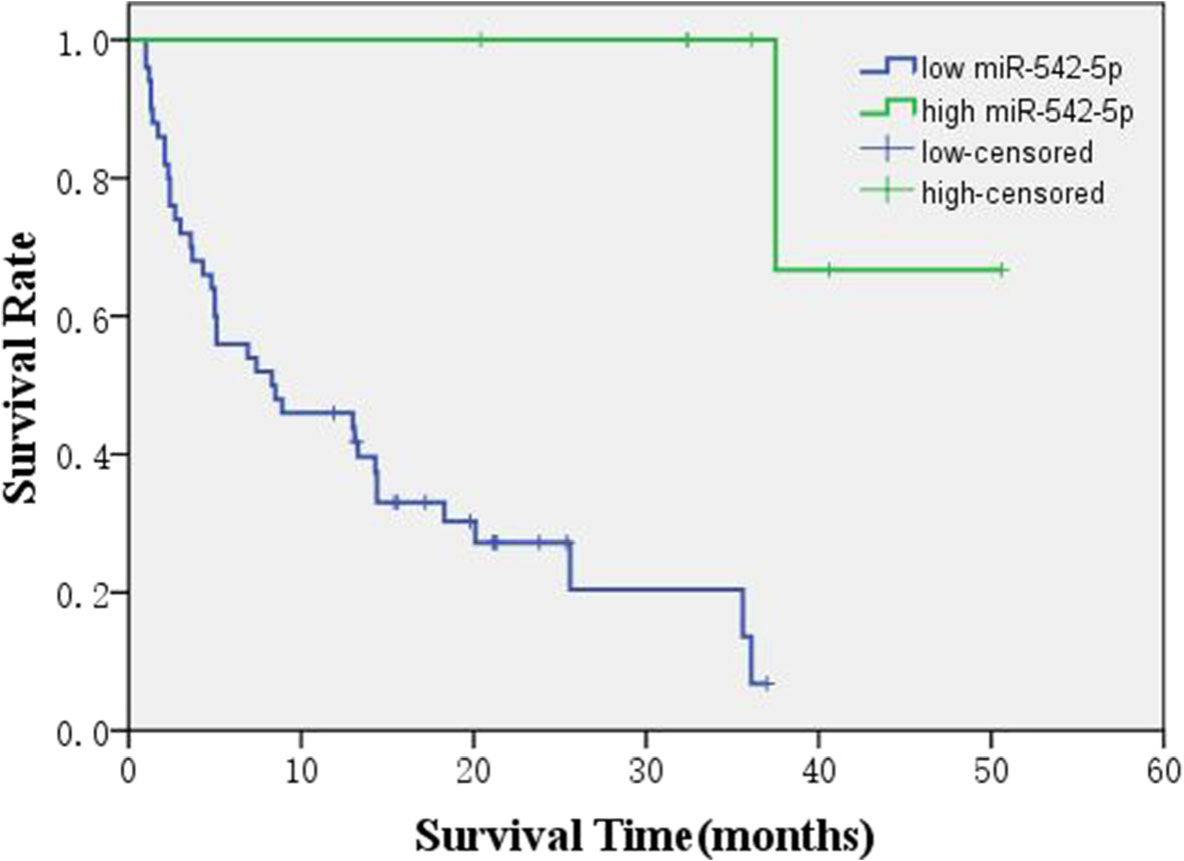
Kaplan-Meier survival curves in 57 patients with lung adenocarcinoma with different miR-542-5p levels

Based on the median expression level of miR-542-5p in NSCLC patients (3.260 ± 2.197), we divided the patients into two groups with high expression of miR-542-5p and low expression of miR-542-5p (4.568 ± 1.993 vs 1.953 ± 1.507). The results of KM curve survival analyses showed that NSCLC patients with lower miR-542-5p expression (*n* = 50, 11.274 ± 1.387 months) had a significantly poorer prognosis than those patients with higher miR-542-5p expression (*n* = 7, 35.714 ± 3.469 months) (*t* = −6.219, p < 0.001, Fig. 3). The multivariate analysis showed that the HR of miR-542-5p was 0.948 (95% CI: 0.916–0.982, *p* = 0.003), which was adjusted by other clinical parameters as gender, age, tumor size, clinical stage, and tumor grading.

### In vivo study using the CAM model

**Fig. 4:**
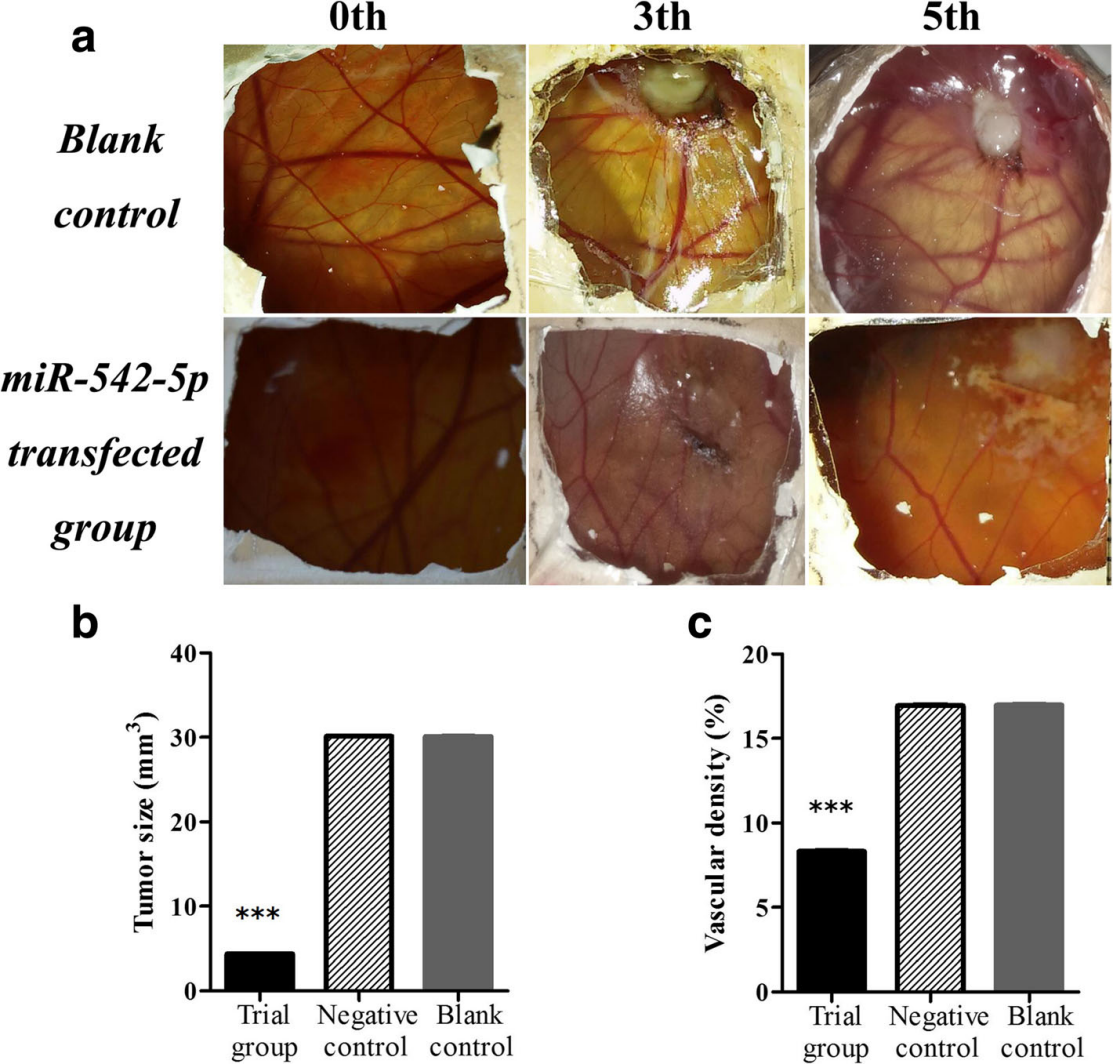
The effect of miR-542-5p on tumor size and angiogenesis in the CAM model. **a**: Selected CAM pictures of a blank control and a miR-542-5p-transfected group on the 0th, 3rd and 5th day of transfection; **b**: Different sizes of transfected tumors on blank control, negative control and miR-542-5p-transfected groups; **c**: Different vascular density on blank control, negative control and miR-542-5p-transfected groups

**Fig. 5:**
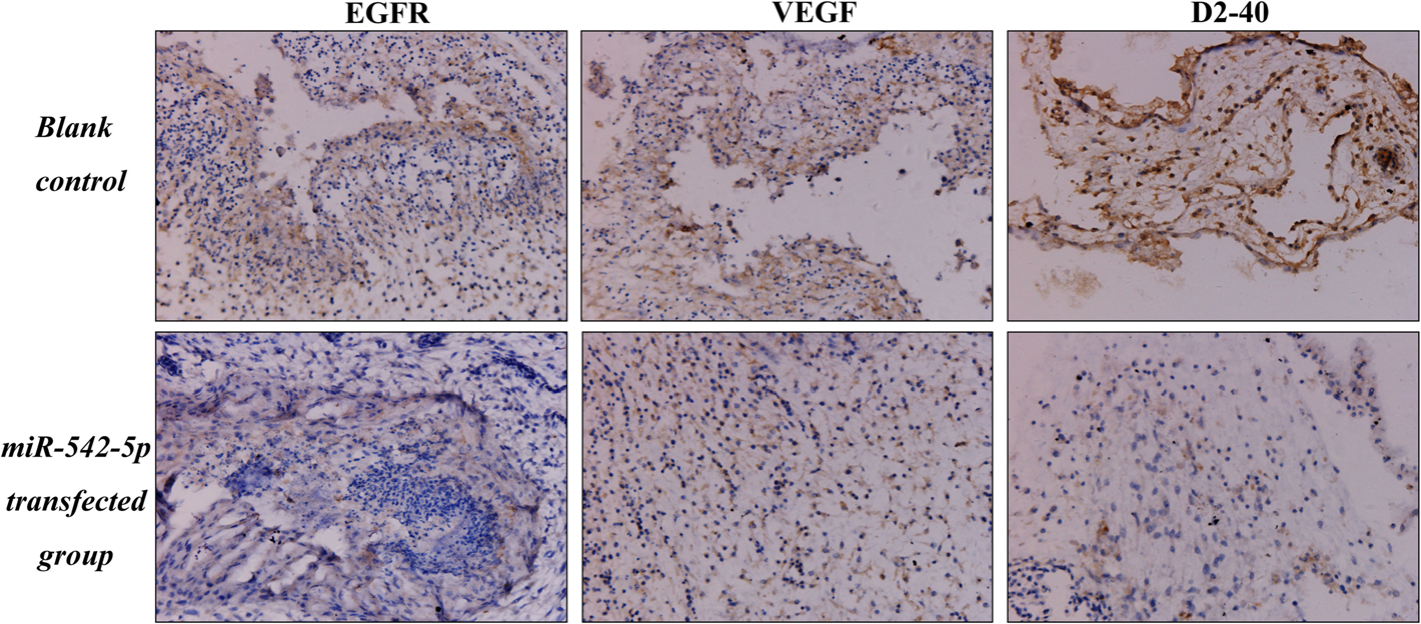
Immunohistochemical staining showing the status of EGFR, VEGF and D2–40 in blank control and miR-542-5p-transfected CAM groups

MiR-542-5p was also detected by RT-qPCR in NSCLC cell lines H460, PC9, H1299 and A549. Among the four different cell lines, the lowest level of miR-542-5p was found in H460, and it was selected for transfection with a miR-542-5p mimic to unveil the function of miR-542-5p in NSCLC. The efficiency of lentiviral transfection was higher than 90%. In CAM, the tumor size was assessed after cells were transplanted and harvested on day 5. Compared to the blank and negative control groups, the trial group showed smaller tumor size, and angiogenesis was suppressed (Fig. 4). The expression levels of EGFR, VEGF and D2–40 in the trial group were weaker, when compared to the blank control group (Fig. 5).

### Potential target genes of miR-542-5p and functional annotation analysis

**Fig. 6:**
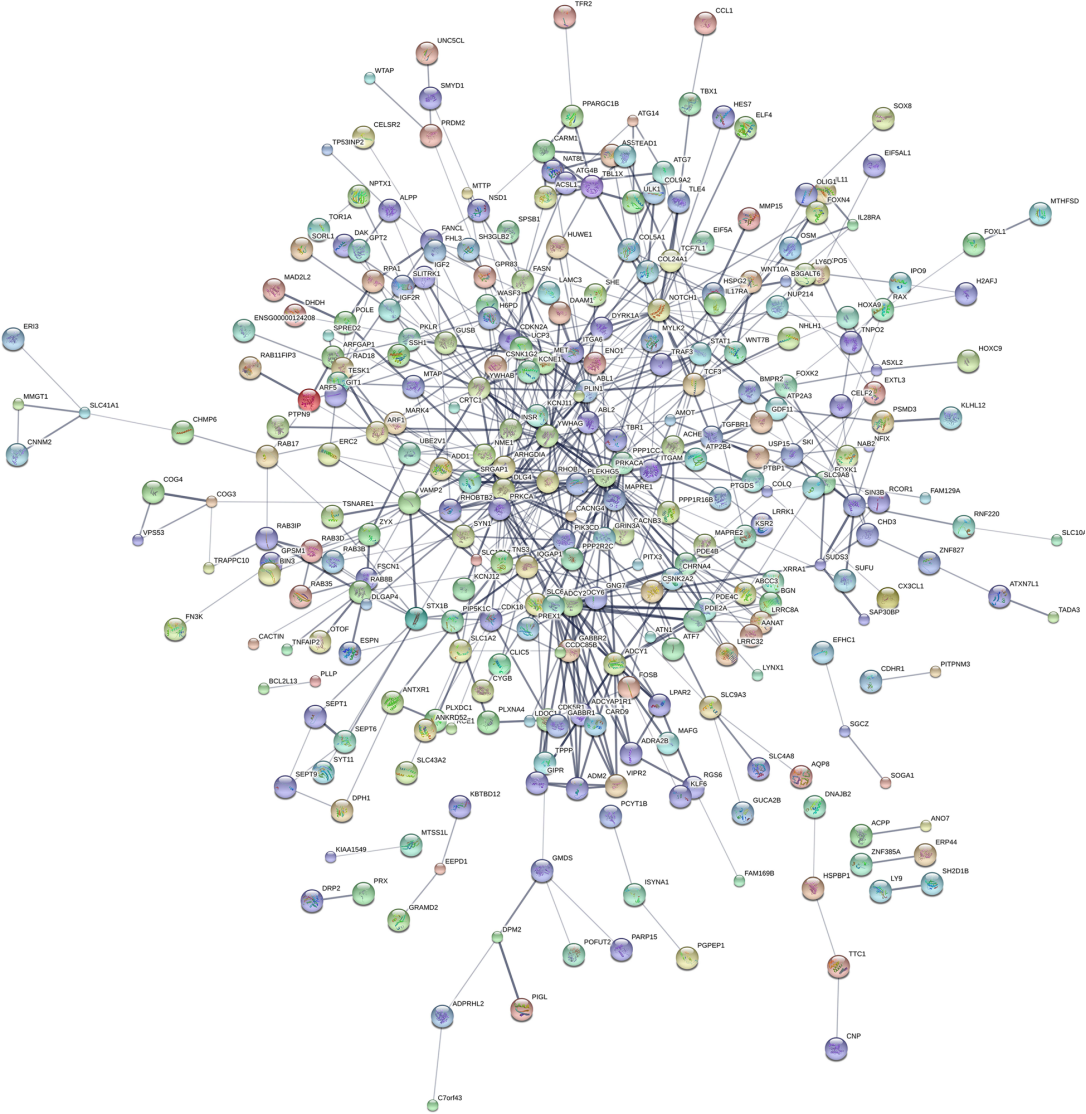
Connections between the predicted target genes of miR-542-5p

**Fig. 7:**
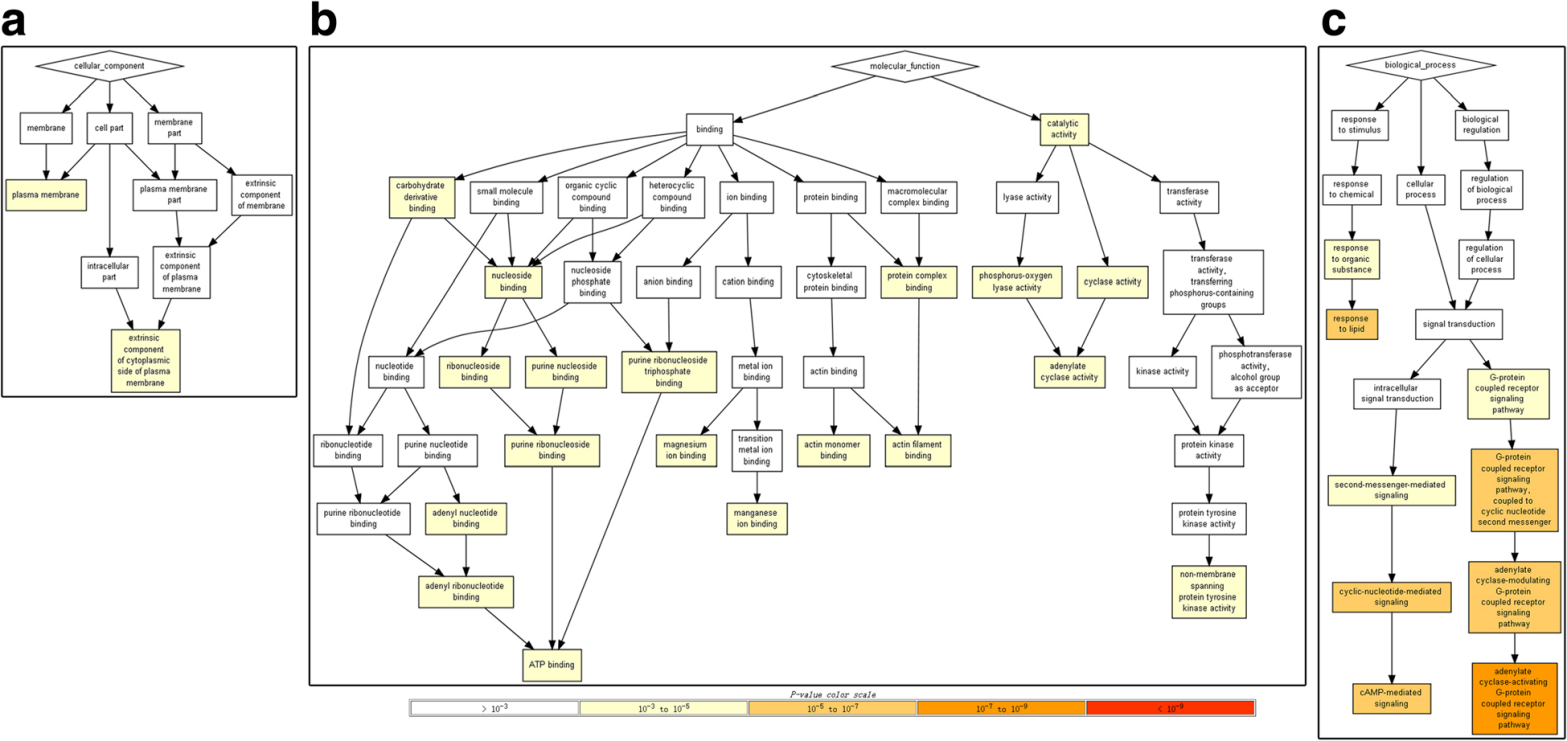
Gene ontology terms enriched by the potential target genes of miR-542-5p. (**a**: Cellular component terms of GO; **b**: Molecular function terms of GO; **c**. Biological process terms of GO)

**Fig. 8:**
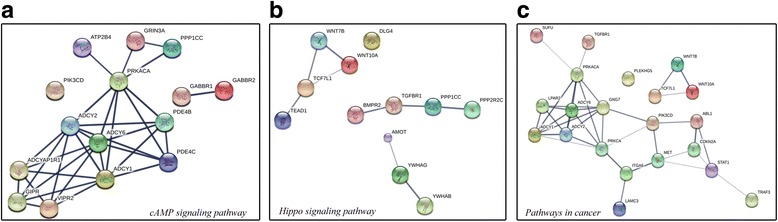
Connection among hub genes predicted on KEGG pathways. **a**. cAMP signaling pathway; **b**. Hippo signaling pathway; **c**. Pathways in cancer

**Fig. 9:**
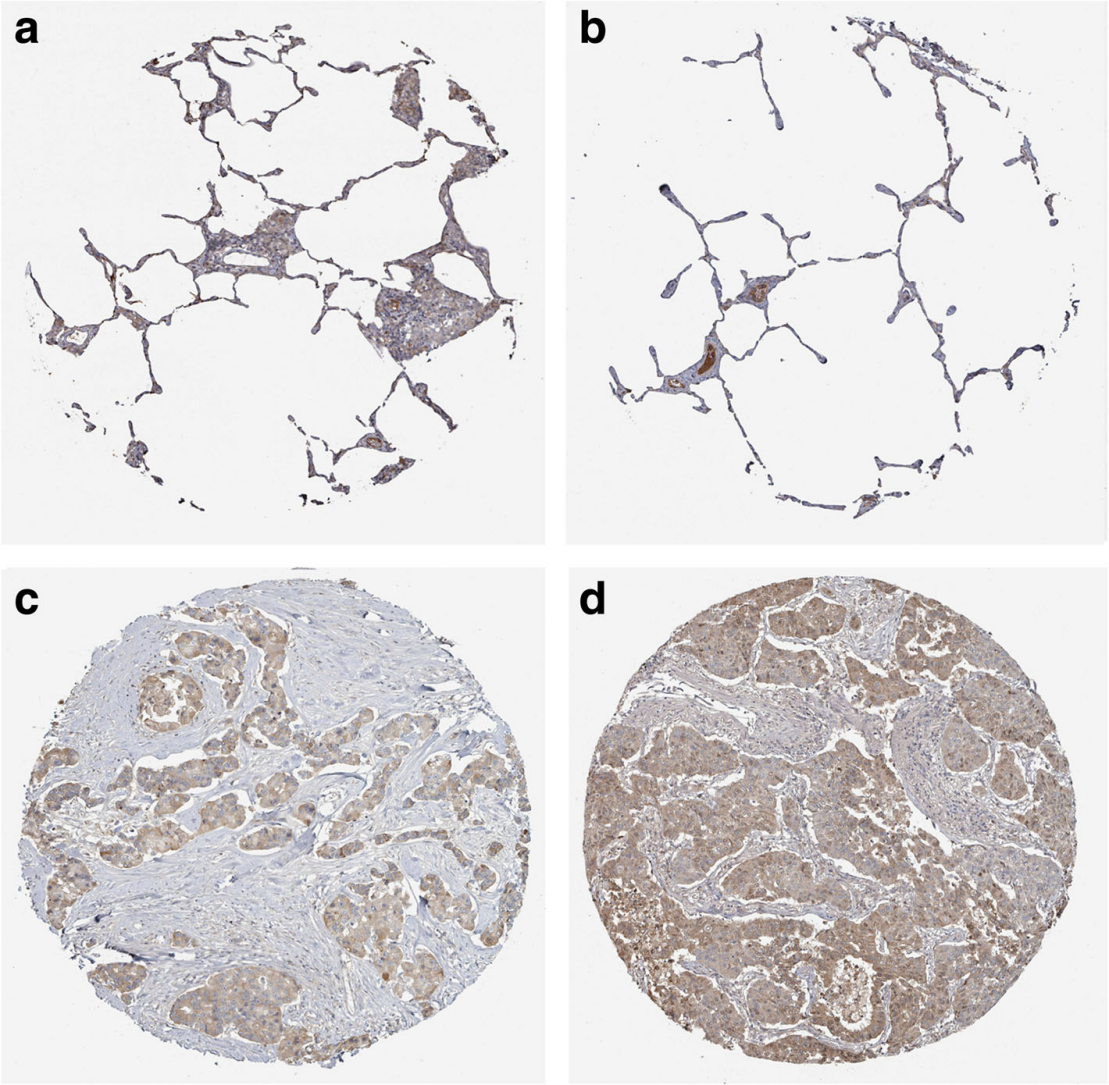
Validation of the protein expression of GABBR1 in NSCLCs. GABBR1 protein was detected by the antibody of HPA050483. **a**, **b**: normal lungs with pneumocytes being not detected. **c**: medium staining in LUAD. **d**: medium staining in LUSC. Immunohistochemistry, ×100

**Fig. 10:**
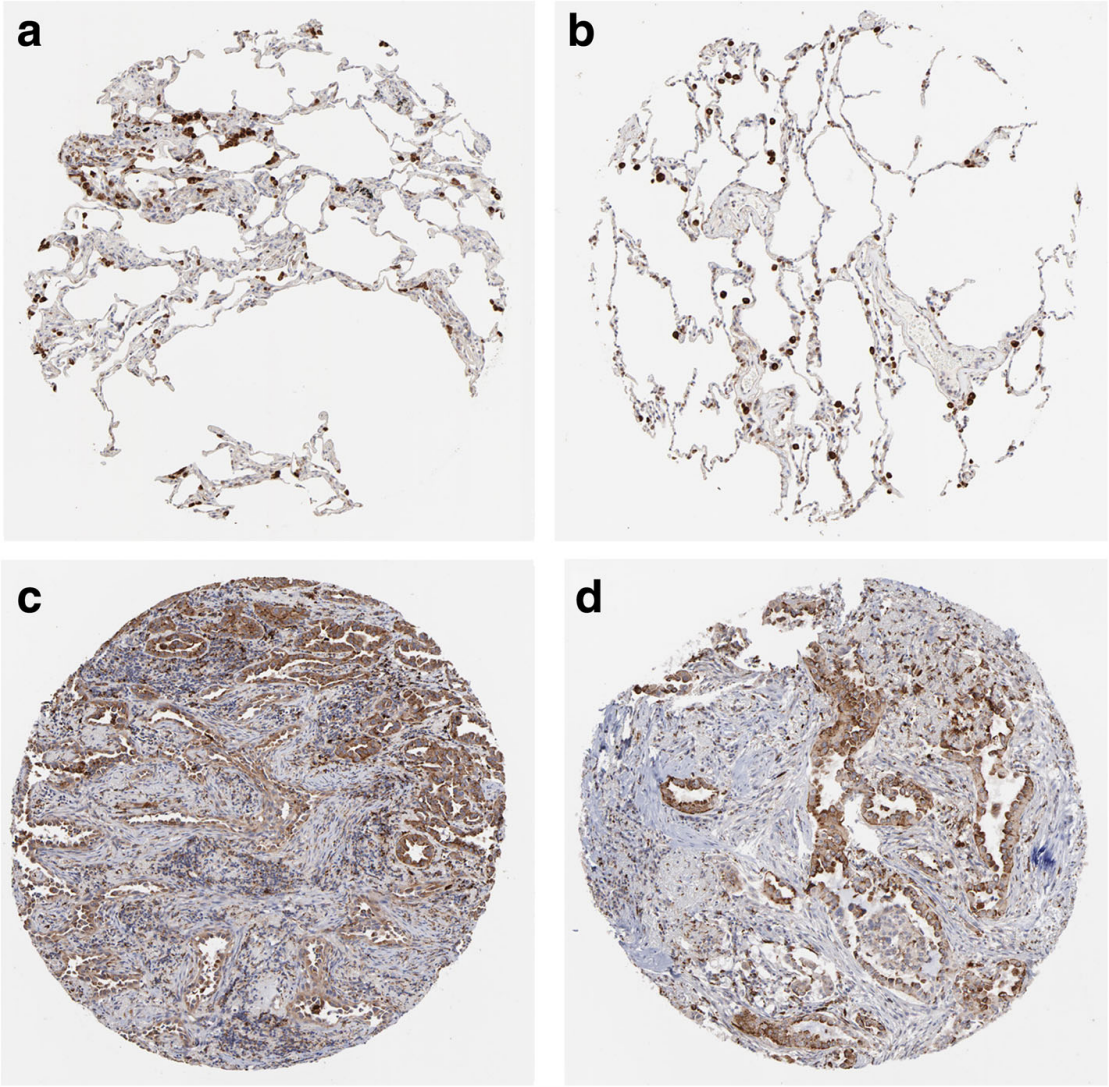
Validation of the protein expression of PDE4B in NSCLCs. PDE4B protein was detected by the antibody of HPA003005. **a**, **b**: pneumocytes in normal lungs with low staining and macrophages with high expression. **c**: medium staining in LUAD. **d**: high staining in LUAD. Immunohistochemistry, ×100

**Fig. 11:**
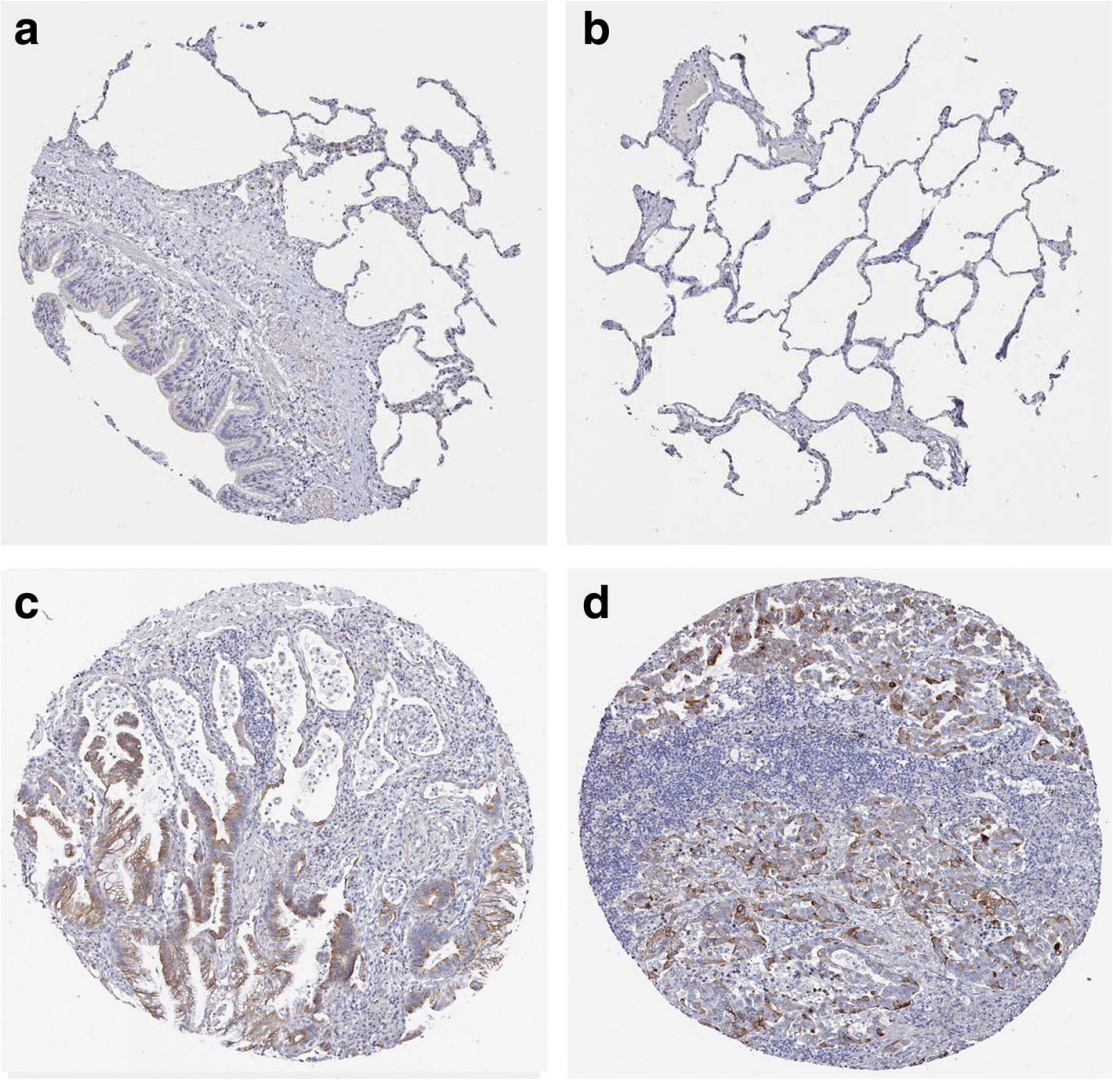
Validation of the protein expression of PDE4C in NSCLCs. PDE4C protein was detected by the antibody of HPA048975. **a**, **b**: pneumocytes in normal lungs with no staining. **c**, **d**: high staining in LUAD. Immunohistochemistry, ×100

**Fig. 12:**
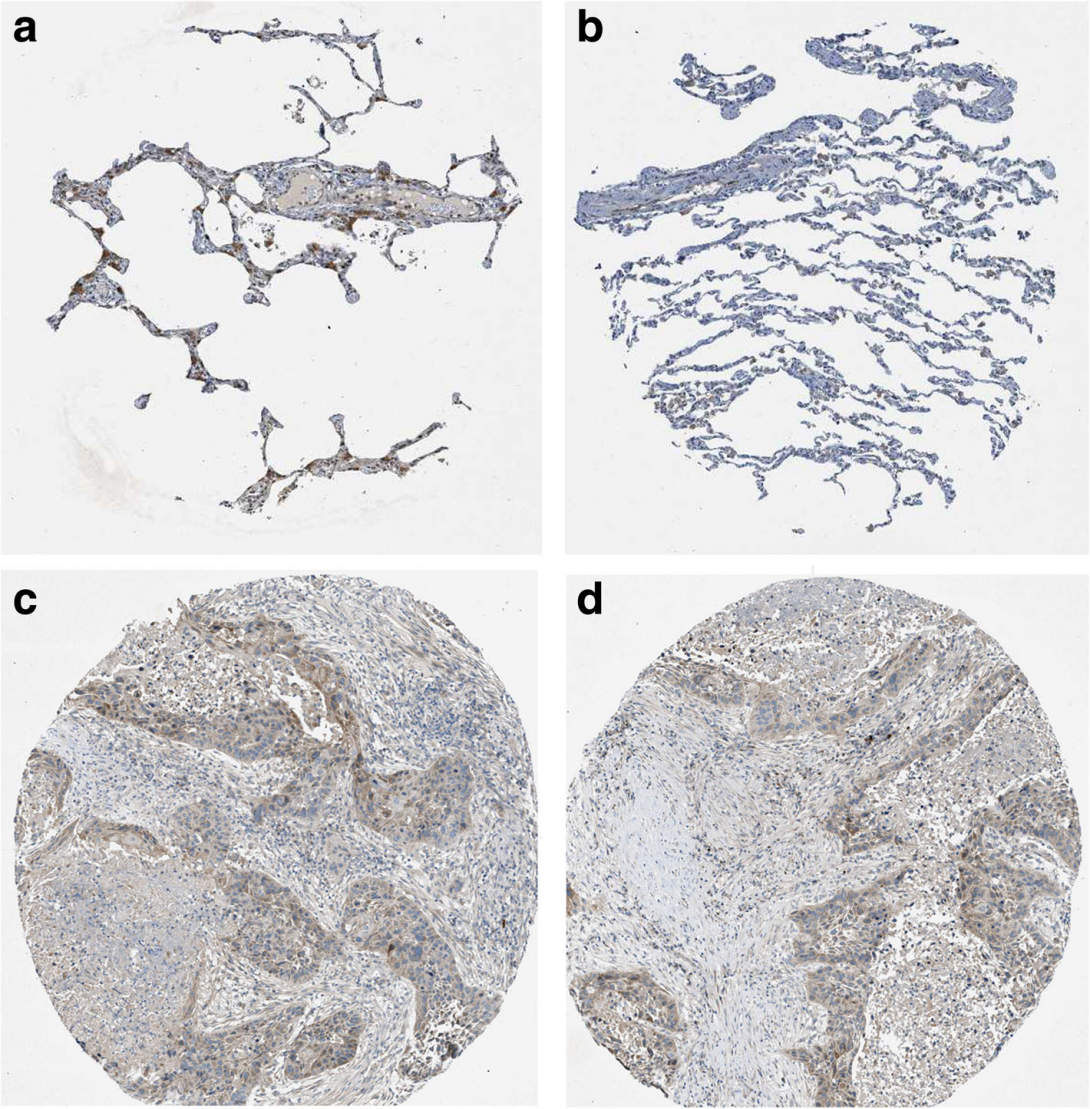
Validation of the protein expression of ADCY6 in NSCLCs. ADCY6 protein was detected by the antibody of CAB018365. **a**, **b**: pneumocytes in normal lungs with no staining. **c**, **d**: medium staining in LUSC. Immunohistochemistry, ×100

**Fig. 13:**
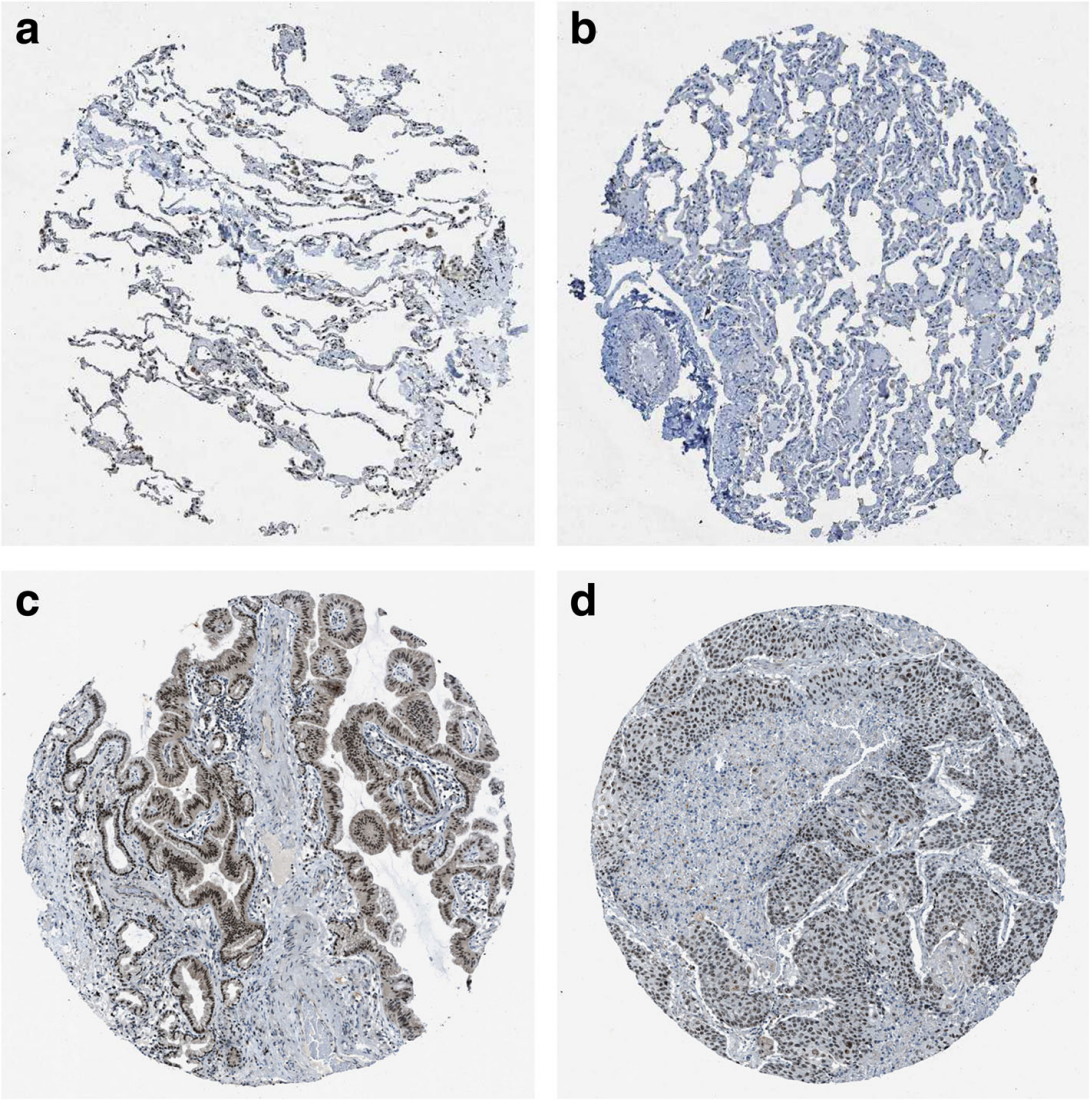
Validation of the protein expression of ADCY1 in NSCLCs. ADCY1 protein was detected by the antibody of CAB018364. **a**, **b**: pneumocytes in normal lungs with no staining, macrophages with medium staining. **c**: high staining in LUAD. **d**: high staining in LUSC. Immunohistochemistry, ×100

**Fig. 14:**
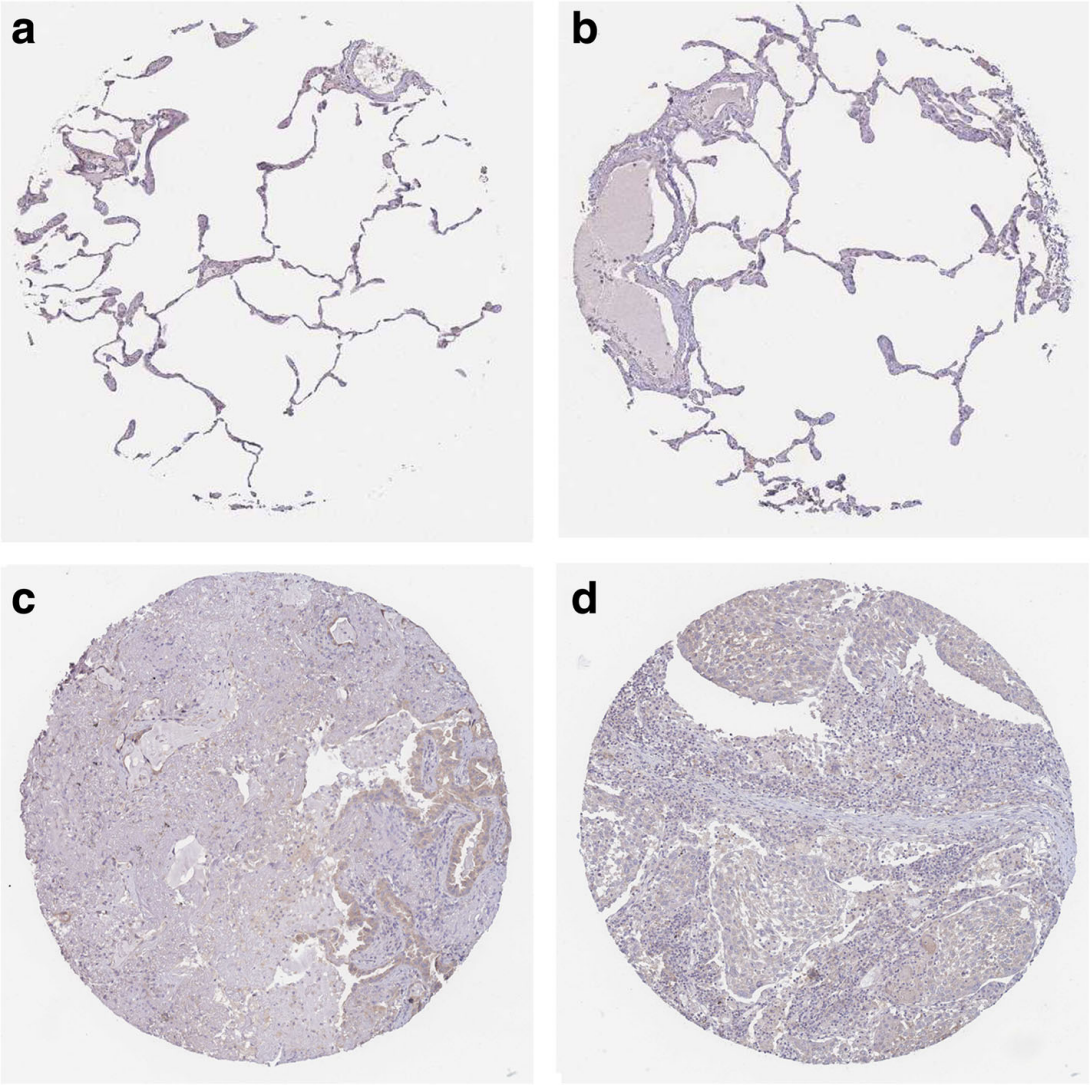
Validation of the protein expression of GIPR in NSCLCs. GIPR protein was detected by the antibody of CAB022710. **a**, **b**: pneumocytes in normal lungs with no staining. **c**: medium staining in LUAD. **d**: low staining in LUSC. Immunohistochemistry, ×100

A total of 457 target genes of miR-542-5p were predicted by the 12 platforms mentioned above. Close connections were found among the genes listed in Fig. 6. These genes were analyzed by GO and KEGG pathways. In the CC of GO analysis, the genes were significantly enriched in the plasma membrane and extrinsic components of the cytoplasmic side of the plasma membrane (Fig. 7a). In MF, non-membrane spanning protein tyrosine kinase activity, ATP binding and other binding items were significantly enriched by genes (Fig. 7b). In BP, the adenylate cyclase-activating G-protein coupled receptor signaling pathway was the most significantly enriched pathway (Fig. 7c). The most significantly enriched KEGG pathway was morphine addiction; and cancer-related mechanisms, such as the cAMP signaling pathway and the Hippo signaling pathway, were also significantly enriched in the 457 potential target genes of miR-542-5p (Table 2, Fig. 8). The genes from cAMP signaling pathway were selected for further protein expression validation in NSCLC tissues by using data from Proteinatlas. Six genes showed particularly stronger expression pattern in lung cancer tissues, as compared to normal lungs (Figs. 9, 10, 11, 12, 13 and 14), which demonstrated that these six genes (GABBR1, PDE4B, PDE4C, ADCY6, ADCY1 and GIPR) had more likelihood of being direct targets of miR-542-5p in lung cancers. However, this hypothesis still needs in vitro and in vivo verification.

**Table 2:** The KEGG pathways enriched by the potential target genes of miR-542-5p

ID	Description	Count in gene set	*p*-value
hsa05032	Morphine addiction	11	0.000111
hsa04024	cAMP signaling pathway	15	0.000587
hsa04261	Adrenergic signaling in cardiomyocytes	12	0.001364
hsa04727	GABAergic synapse	9	0.001523
hsa05030	Cocaine addiction	7	0.001541
hsa04390	Hippo signaling pathway	12	0.001790
hsa04725	Cholinergic synapse	10	0.002243
hsa04724	Glutamatergic synapse	10	0.002693
hsa05200	Pathways in cancer	21	0.002768
hsa04923	Regulation of lipolysis in adipocytes	7	0.003081
hsa05414	Dilated cardiomyopathy	8	0.005886
hsa04911	Insulin secretion	8	0.006278
hsa04921	Oxytocin signaling pathway	11	0.007747
hsa04976	Bile secretion	7	0.008632
hsa04520	Adherens junction	7	0.009886

Enriched by DAVID, *p* < 0.01

## Discussion

In this study, miR-542-5p was found to be predominantly down-regulated in NSCLC tissues, which had a negative effect on the prognosis of NSCLC patients. Exploration of the effect of miR-542-5p on NSCLC with a CAM model confirmed the suppressive function of miR-542-5p on cell growth and angiogenesis of NSCLC. Furthermore, the mechanism of miR-542-5p predicted by bioinformatical approaches suggested valuable pathways that might relate to tumorigenesis and tumor development.

Many dysregulated miRNAs have been shown to relate to tumorigenesis or development in numerous cancers. In published studies, miR-542-5p was described as a tumor-suppressed miRNA in endometrial carcinosarcoma [14], neuroblastoma [15] and rectal cancer [16]. However, in several cancers, miR-542-5p was regarded as a cancer promotor. For example, in osteosarcoma, miR-542-5p was overexpressed in cancer tissues and linked with poor prognosis [17]. In lung cancer, the effect of miR-542-5p was reported in only one publication [18]. In the study of Yamaguchi et al. [18], miR-542-5p was found to be inversely expressed with EGFR in lung cancer tissues when tested by immunohistochemistry, which was consistent with our study. In addition, the expression of VEGF and D2–40 in NSCLC tissues were also tested in current study, and both of them were supported the idea that miR-542-5p can suppress angiogenesis in NSCLC. In vitro, miR-542-5p was reported to suppress the proliferation of the lung cancer cell line A549, which was supported by our CAM assays. In the study of Yamaguchi et al. [18], because of the lack of normal lung tissues to compare with, the relative level of miR-542-5p in lung cancer tissues was not clear. To supplement this research, the differential expression of miR-542-5p between NSCLC and adjacent normal lung tissues was tested in the current study, and the results suggested that miR-542-5p was notably down-regulated in NSCLC tissues. It is also interesting to find that the lower miR-542-5p level in NSCLC could predict the poorer prognosis with the adjust HR being 0.948 (*p* = 0.003), which further confirms that miR-542-5p acts as a tumor-suppressive miRNA in the pathogenesis and progression of NSCLC, and higher level of miR-542-5p level could act as a protective indicator of NSCLC. However, this finding needs to be verified with larger sample size.

Although many miRNAs have been affirmed as tumor suppressors or promoters in NSCLC, the functional mechanism of miRNAs in NSCLC was still unclear. In the current study, we explored the potential target genes of miR-542-5p using 13 programs with different algorithms, then analyzed target genes by functional annotation. The enriched results of GO analysis and KEGG pathways suggested that most potential target genes are significantly related to message transfer. The trio of enriched KEGG pathways, the cAMP signaling pathway, the Hippo signaling pathway, and other cancer-related pathways, hinted at the probable mechanism of miR-542-5p in cancers. Cyclic adenosine monophosphate (cAMP), as a second messenger, can regulate cellular responses by activated effectors [19]. The most famous effector of cAMP is the cAMP-dependent protein kinase (PKA). Shaikh et al. [20] found that prevention of the activity of PKA could suppress the hypoxia-mediated epithelial-mesenchymal transition (EMT), which is involved in invasion and migration in human lung cancer cells. cAMP signaling can also down-regulate p300, which is a transcriptional coactivator, through Epac and p38 MARK [21]. The Hippo signaling pathway was another significant enriched KEGG pathway found in our analysis. It has been reported to regulate the proliferation and apoptosis of cells, mediated by transcription coactivators like yes-associated protein (YAP) [22]. Several studies of the Hippo signaling pathway in NSCLC have been published [23–25]. You et al. [23] found that Hippo/YAP signaling was inhibited after knockdown of ERK1/2. In breast cancer cells, Zhang et al. [26] found that the Hippo signaling pathway has an effect on EMT. EMT is also a vital process in NSCLC, which is developed from normal lung epithelial cells [27]. Wnt signaling was reported to relate to Hippo signaling [28], and it was notable that two members of the Wnt family (Wnt10A and Wnt7B) were predicted as target genes of miR-542-5p in the current study and were enriched in the Hippo signaling pathway. In our functional annotation of genes, the cAMP and Hippo signaling pathways were enriched in 15 and 12 genes, respectively. To test the predicting power and validate the potential target genes of miR-542-5p in NSCLC, the protein level of all the genes involved in the cAMP pathway were checked in Proteinatlas. Interestingly, six genes (GABBR1, PDE4B, PDE4C, ADCY6, ADCY1 and GIPR) were confirmed to be overexpressed in NSCLCs tissues. These six genes have greater possibility to be real target genes of miR-542-5p in NSCLCs. This signaling pathway might play an integral part in the potential mechanism of miR-542-5p in NSCLC.

## Conclusions

In conclusion, the current study suggests that miR-542-5p acts as an anti-oncogene in NSCLC and that the cAMP and Hippo signaling pathways may be the most likely mechanisms regulated by miR-542-5p. However, because of the limited amount of included tissues and lack of further investigation in vitro or of the mechanism of action of miR-542-5p in NSCLC, more researches are needed to clarify the effect of miR-542-5p on NSCLC in the future.

## Abbreviations

CAM: Chick chorioallantoic membrane
cAMP: cyclic adenosine monophosphate
EMT: Epithelial-mesenchymal transition
FFPE: Formalin-fixed and paraffin-embedded
GO: Gene ontology
HE: Hematoxylin and eosin
HRs: Hazard ratios
IHC: Immunohistochemical
KEGG: Kyoto encyclopedia of genes and genomes
KM: Kaplan-Meier
LUAD: Lung adenocarcinoma
LUSC: Lung squamous cell carcinoma
miRs: microRNAs
NSCLC: Non-small cell lung cancer
PKA: Protein kinase
qRT-PCR: Quantitative reverse transcriptase-polymerase chain reactions
YAP: Yes-associated protein
